# Longitudinal Measurement Invariance of the Harmonized Cognitive Assessment Protocol in Mex-Cog

**DOI:** 10.1093/geroni/igaf122.3974

**Published:** 2025-12-31

**Authors:** Sangha Jeon, Zachary Kunicki, Lily Kamalyan, Sarah Prieto, Rebeca Wong, Miguel Rentería, Emily Briceño

**Affiliations:** University of Michigan, Ann Arbor, Ann Arbor, Michigan, United States; Warren Alpert Medical of Brown University, Providence, Rhode Island, United States; Taub Institute for Research on Alzheimer’s Disease and the Aging Brain, Columbia University College of Physicians and Surgeons, New York, New York, United States; Brown University, Providence, Rhode Island, United States; University of Texas Health San Antonio, San Antonio, Texas, United States; Taub Institute for Research on Alzheimer’s Disease and the Aging Brain, Columbia University College of Physicians and Surgeons, New York, New York, United States; University of Michigan Medical School, Ann Arbor, Michigan, United States

## Abstract

With the projected increase of older adults with Alzheimer’s disease and Alzheimer’s disease-related dementias (AD/ADRD) in Mexico, there is a growing need for consistent measurement of cognitive functioning over time. Ensuring measurement invariance is critical so that observed changes reflect true cognitive decline or improvement, rather than differences in how the test functions across time. This study evaluated the longitudinal measurement invariance (MI) of the Harmonized Cognitive Assessment Protocol (HCAP) from the Mexican Cognitive Aging Ancillary Study (Mex-Cog). Participants were 1,372 adults aged 55 and older who completed both Wave 1 (2016) and Wave 2 (2021) assessments. We evaluated longitudinal invariance using unidimensional models for five domains (orientation, language, memory, executive functioning, and visuospatial functioning), and a separate higher-order model representing general cognitive performance (GCP). All domain-specific models showed good fit (comparative fit index [CFI] > .95), and the higher-order model fit was acceptable (CFI = .943), with good to acceptable RMSEA (.000-.077) and SRMR (.00-.080). Longitudinal invariance analyses yielded mixed results across cognitive domains: language met only configural invariance, memory showed metric invariance, orientation and executive function showed partial metric invariance, and visuospatial functioning achieved scalar invariance. Results suggest that the factor structure of cognitive domains in Mex-Cog remains stable over 5 years. However, some items functioned differently over time, possibly due to practice effects, indicating that observed changes in these items may not solely reflect true cognitive change. This highlights the importance of examining the longitudinal consistency of cognitive scores over time in population-based cognitive aging studies.

